# Spelling ability influences early letter encoding during reading: Evidence from return-sweep eye movements

**DOI:** 10.1177/1747021820949150

**Published:** 2020-08-25

**Authors:** Adam J Parker, Timothy J Slattery

**Affiliations:** 1 Department of Experimental Psychology, University of Oxford, Oxford, UK; 2 Department of Psychology, Faculty of Science & Technology, Bournemouth University, Poole, UK

**Keywords:** Eye movements, reading, return-sweeps, individual differences

## Abstract

In recent years, there has been an increase in research concerning individual differences in readers’ eye movements. However, this body of work is almost exclusively concerned with the reading of single-line texts. While spelling and reading ability have been reported to influence saccade targeting and fixation times during intra-line reading, where upcoming words are available for parafoveal processing, it is unclear how these variables affect fixations adjacent to return-sweeps. We, therefore, examined the influence of spelling and reading ability on return-sweep and corrective saccade parameters for 120 participants engaged in multiline text reading. Less-skilled readers and spellers tended to launch their return-sweeps closer to the end of the line, prefer a viewing location closer to the start of the next, and made more return-sweep undershoot errors. We additionally report several skill-related differences in readers’ fixation durations across multiline texts. Reading ability influenced all fixations except those resulting from return-sweep error. In contrast, spelling ability influenced only those fixations following accurate return-sweeps—where parafoveal processing was not possible prior to fixation. This stands in contrasts to an established body of work where fixation durations are related to reading but not spelling ability. These results indicate that lexical quality shapes the rate at which readers access meaning from the text by enhancing early letter encoding, and influences saccade targeting even in the absence of parafoveal target information.

Until recently, there has been a paucity of research investigating individual differences in readers’ eye movements. This likely stems from the general assumption that skilled readers read in the same way. This “uniformity assumption” (Andrews, 2012) has led many psycholinguists to average data over readers, thus masking subtle variations in skilled readers’ patterns of saccades and fixations. However, a now substantial body of literature has shown that the eye movement patterns of highly skilled readers are quantitatively different to those of less-skilled readers (Ashby et al., 2005; Bélanger et al., 2012; Drieghe et al., 2019; Kuperman & Van Dyke, 2011; Rayner et al., 2010; Slattery & Yates, 2018; Veldre & Andrews, 2014, 2015; Veldre et al., 2017). Generally, high reading ability is associated with shorter fixation times, while high spelling ability is selectively associated with measures of word skipping (Slattery & Yates, 2018; Veldre & Andrews, 2015, 2016; Veldre et al., 2017). Given that word skipping is dependent on the processing of information in the parafovea (the portion of the retina spanning 2°–5° of visual angle from fixation), this finding suggests that the precise lexical representations indexed by high spelling ability specifically enhance the parafoveal processing (or extraction of parafoveal information) that contributes to oculomotor decisions. Yet, it is unclear how spelling ability influences oculomotor decisions when the target of the saccade is beyond parafoveal vision. Return-sweep saccades (eye movements that take a readers’ fixation from the end of one line to the start of the next) are a common case where readers must target a portion of the text that is beyond parafoveal vision. As with individual differences in eye movement control during reading, return-sweep research is somewhat scarce. Thus, the present experiment examines return-sweep eye movements and explores the role that spelling and reading skill play in their execution. The influence of spelling and reading ability on fixation durations adjacent to return-sweeps is additionally examined. Together this work contributes to these two rapidly developing areas of eye movement reading research.

To reiterate, return-sweeps are saccadic eye movements that are essential for readers to encode passages of written text. While there is variability in launch and landing positions of return-sweeps, line-final (those prior to a return-sweep) and line-initial (those following a return-sweep) fixations tend to fall five to seven characters away from the extremes of the line for skilled readers (Parker, Nikolova, et al., 2019; Parker, Slattery, & Kirkby, 2019; Slattery & Vasilev, 2019). Line-initial fixations can be divided into two subgroups: accurate and undersweep fixations. What differentiates these two types of line-initial fixations is the direction in which they move next. Accurate line-initial fixations are those that land close enough to the target of the return-sweep and are followed by a progressive rightward movement through the text. Undersweep fixations are those that are followed by an immediate leftward corrective saccade prior to the readers’ rightward pass. Undersweep fixations are generally considered to result from oculomotor error. Return-sweep fixations tend to differ in duration from intra-line fixations that are non-adjacent to return-sweeps (Parker, Nikolova, et al., 2019; Parker, Slattery, & Kirkby, 2019). While accurate line-initial fixations tend to be longer than intra-line fixations, both line-final and undersweep fixations tend to be shorter. As will be become clear, there is evidence to argue for differential contributions of each fixation population to ongoing visual oculomotor and linguistic processing. To foreshadow, these differences will contribute to the specific predictions we make concerning measures of spelling and reading ability for the duration of each fixation population.

Kuperman et al. (2010) reported that fixation times during paragraph reading are influenced by visual boundaries of the text, whereby intra-line fixations show a decrease in duration that varies in relation to a word’s ordinal position on the line. This was argued to be independent of lexical, contextual, and oculomotor predictors of eye movement behaviour. Consistent with these speed-up effects, line-final fixations were shorter than those occurring intra-line. Kuperman et al. (2010) argued that these reductions reflect the processing of line breaks and return-sweep planning. Consistent with this planning account, Hofmeister (1997) reported that text degradation of 50% led to a 20-ms increase in duration for all fixations except line-final fixations. At the very least, this indicates that line-final fixations are less influenced by stimulus quality. In contrast, accurate line-initial fixations are longer than intra-line fixations (Parker, Nikolova, et al., 2019; Parker, Slattery, & Kirkby, 2019) and have been argued to be the consequence of a lack of parafoveal preview (Parker et al., 2017; Parker, Nikolova, et al., 2019). Finally, undersweep fixations typically result from oculomotor error with longer lines yielding more corrective saccades (Heller, 1982; Hofmeister et al., 1999; Parker, Nikolova, et al., 2019). While their duration is uninfluenced by lexical properties of the fixated word (Slattery & Parker, 2019), there is now converging evidence to suggest that readers are able to extract information at the point of the undersweep fixation (Parker et al., 2020; Slattery & Parker, 2019) and to the left of undersweep fixations (i.e., the line-initial word; Parker et al., 2020; Parker & Slattery, 2019). Given the hypothesised differential involvement of lexical processing for each population of fixation, there may be differences in the extent to which lexical quality influences the duration of each fixation population.

In terms of skill-related differences in return-sweep and corrective saccade parameters, research has focused on the frequency of corrective saccades following a return-sweep. Generally, these findings are consistent in showing that less-skilled adult readers (i.e., those who performed poorly on a word identification task; Heller, 1982), typically developing (Netchine et al., 1983), and dyslexic children (Trauzettel et al., 2010) require more corrective saccades following a return-sweep to reach the left margin. This leads to the conclusion that less-skilled readers require additional corrective saccades to reach the target of the return-sweep. These findings are highly consistent with data presented by Parker, Slattery, and Kirkby (2019) who reported that adults and children require corrective saccades following 51.5% and 62.4% of return-sweeps, respectively. Parker, Slattery, and Kirkby additionally provided a thorough investigation of return-sweep and corrective saccade parameters in both adults and children. It was reported that, compared with adults, children’s return-sweeps were launched closer to the end of the line and landed closer to the start. The authors concluded that less-skilled child readers fixate on more extreme positions on the line to encode words in foveal vision. If reading skill and lexical quality are linked to rates of foveal and parafoveal encoding, then we would expect less-skilled readers to similarly fixate these more extreme positions.

Lexical quality refers to the precision and redundancy of readers’ orthographic representations and coherent connections to associated phonological and semantic information (Perfetti, 2007). High-quality representations afford rapid, automatic lexical retrieval that allows readers to devote their limited attentional resources to comprehension processes (Perfetti, 2007). Investigations of lexical quality have often relied on measures of reading comprehension and vocabulary, like those used by Jared et al. (1999) who found that more skilled readers, as indexed by the comprehension section of the Nelson–Denny Reading Test, made significantly shorter fixations than did poorer readers. Furthermore, for the processing of non-predictable low-frequency words, poorer readers relied more on phonological activation than better readers, highlighting how reading skill influences word identification when words are particularly difficult to process. Although Jared et al. reported that phonological activation influences foveal processing under certain conditions, this stands in contrast to research looking at parafoveal processing of phonology which reports that less-skilled readers do not extract phonological codes in parafoveal vision (e.g., Chace, Rayner, & Well, 2005; Vasilev, Yates, & Slattery, 2019). Ashby et al. (2005) reported differences in the reading strategies employed by good and poor readers (again indexed by the Nelson–Denny scores), where better readers relied less on context to support word processing. This illustrates that highly skilled reading relies on rapid, autonomous lexical retrieval processes that place little reliance on context for word identification (Perfetti, 1992; Stanovich, 2000). Consistent with this, Kuperman and Van Dyke (2011) noted that word identification and tests of rapid letter and digit naming were the most robust predictors of fixation duration measures.

Andrews (2012) argued that, while vocabulary and comprehension are useful indices of the efficiency of lexical and semantic retrieval, they need to be complemented by measures of spelling ability to capture the precise, word-specific knowledge that is central to Perfetti’s (2007) specification of lexical quality. Consistent with the view that reading comprehension and spelling ability tap different components of lexical quality, Veldre and Andrews (2015, 2016, see also Drieghe et al., 2019; Slattery & Yates, 2018; Veldre et al., 2017) have demonstrated differential effects of these two aspects of proficiency on skipping probabilities and fixation durations. Specifically, higher skipping percentages and longer saccade lengths are associated almost exclusively with high spelling ability, whereas high reading ability is primarily associated with faster reading times. One interpretation of these dissociated effects would be that reading ability influences foveal processing while spelling ability influences parafoveal processing. However, another possibility is that spelling ability influences early orthographic encoding regardless of whether this occurs foveally or parafoveally while reading ability influences later lexical processing and the decision to move the eyes to a new word. Still, another interpretation would be that better spellers have learned to adopt a riskier reading strategy that relies on longer saccades (Rayner et al., 2006). So, it is possible that return-sweep saccades will also be influenced by a reader’s spelling ability. However, since return-sweeps travel much further than typical saccades, their target will lie far outside parafoveal vision. Therefore, it is unclear if and how lexical quality may affect return-sweep saccades. This is the focus of this study.

With regard to how our measures of lexical quality will influence our eye movement measures, we made several predictions. Our first aim was to investigate how return-sweep parameters (launch site, landing site, and frequency of corrective saccades) are influenced by measures of lexical quality. Parker, Slattery, and Kirkby (2019) reported that relative to skilled adult readers, children launched their return-sweeps closer to the end of the line and targeted a location closer to the start of the next. These effects have been hypothesised to reflect developing readers’ tendency to rely on foveal encoding in these locations. Of interest to the current study then are the results reported by Veldre and Andrews (2014). Veldre and Andrews reported that high reading and spelling ability were both associated with increased use of information in parafoveal vision to the right of fixation (but interestingly not the left). Furthermore, the best readers and spellers were most disrupted when denied close parafoveal information. If, as indicated by these results, lexical quality influences the amount of information encoded in the perceptual span (i.e., the area from which readers obtain useful information), it is expected that the line-final fixations of readers with lower quality lexical representations would occur closer to the end of the line as these readers cannot rely on parafoveal processing of this information.

Similarly, return-sweep targeting may vary as a function of lexical quality. For instance, better spellers may have developed an optimal strategy that involves targeting further into a new line since they will be more capable of encoding letters that are further from fixation than poor spellers. This is consistent with a recent report that readers adjust the targeting of their return-sweeps to meet the typographic environment (Vasilev, Adedeji, et al., 2019). Vasilev et al. had readers read blocks of either small or large font stimuli. They found that with text displayed in a smaller font, readers adjusted their return-sweeps to land closer (in visual angle) to the start of the new line. They argued that this strategy prevented readers from landing in a position with too many letters to the left of their line-initial fixation. If individual readers can learn to adjust their return-sweep targeting based on low-level typographic properties in the span of a 60-minute experiment, then it seems likely that they would be able to learn to do this based on their ability to encode words which they have developed over many years of reading. Given reports of increased corrective saccades in less-skilled readers (e.g., Parker, Slattery, & Kirkby, 2019), it is expected that those with lower quality lexical representations will require additional corrective saccades to reach the start of the line. This prediction follows from the notion that less-skilled readers target closer to the start of a new line and launch their return-sweeps from closer to the end of the prior line due to encoding limitations. That is, longer intended saccades will be more influenced by saccadic error.

An increase in corrective saccades for less-skilled readers/spellers may also be due to a reduced ability to parafoveally encode letters. It is important to note that this prediction assumes that there is a leftward skew of attention when readers make return-sweeps (i.e., leftward saccades). This assumption is consistent with the notion that the movement of attention is consistent with the direction of the saccade that it precedes (Godijn & Pratt, 2002; Godijn & Theeuwes, 2003), and the observation that readers acquire information from the line-initial word when their line-initial fixation lands to its right (e.g., Parker et al., 2020). Of course, this is an open question as Veldre and Andrews (2014) reported that spelling ability did not influence the extraction of information to the left of rightwards moving intra-line fixations. An alternative explanation for an increase in corrective saccades for poorer readers/spellers is that better readers and/or spellers could adopt a risky reading strategy (McGowan & Reichle, 2018; Rayner et al., 2006). Such a strategy might predict that readers learn they can target their return-sweeps farther into the next line and avoid corrective saccades that fixate the beginning of lines (i.e., riskier reading).

Our second aim was to investigate how measures of lexical quality influenced return-sweep fixations (line-final, accurate line-initial, undersweep). Line-final fixations have been hypothesised to be involved in return-sweep planning (e.g., Hofmeister, 1997) rather than word processing. Therefore, these fixations may not be under direct lexical control and measures of lexical quality may not influence these fixation durations. Instead, they may be under oculomotor control. The same reasoning holds for undersweep fixations. These short fixations are assumed to be terminated by the automatic triggering of a corrective saccade when the reader lands in a non-efficient or unattended location (Heller, 1982). However, for fixations that follow accurate return-sweeps, lexical quality should play a role as these fixations are on words that received no parafoveal processing and thus all the work of word identification must happen in foveal vision. Furthermore, these accurate line-initial fixations are useful for determining if spelling ability is associated with parafoveal processing in the form of trans-saccadic integration or is instead associated with an initial stage of orthographic encoding. During intra-line reading, initial orthographic encoding is likely to take place during parafoveal processing and so it is impossible to sort out these two explanations for the influence of spelling ability. However, prior to a return-sweep, there can be no parafoveal processing of words at the start of the next line. Therefore, any trans-saccadic integration would be severely limited and unlikely to influence line-initial fixation durations. However, if spelling ability influences early orthographic encoding, then we should see evidence of this on the duration of accurate line-initial fixations as this early encoding could not have happened on the prior fixation.

These predictions were examined in an eye movement experiment of multiline reading where line length was manipulated. This manipulation allowed us to additionally examine the influence of text layout on eye movement control during the processing of multiline texts which was an interest of early investigations of return-sweep saccades (Heller, 1982; Hofmeister et al., 1999). To foreshadow, the inclusion of this manipulation enabled us to replicate several effects reported in the literature thus lending strength to our novel findings.

## Method

### Participants

A total of 123 native English speakers from the Bournemouth University community participated in the study. All had normal or corrected-to-normal vision and indicated that they had no history of reading impairment. Two participants were excluded due to track loss and one excluded to due below chance performance (<50% accuracy) on comprehension items. This left 120 participants, with a mean age of 23.4 years (*SD* = 10.28), who were naïve to the purpose of the experiment.

### Apparatus

Eye movements were recorded via an SR Research EyeLink 1000 eye-tracker sampling once per millisecond. Although reading was binocular, monocular data were recorded. The right eye was tracked for all but four participants.^1^ Text was presented in black letters on a white background using a non-proportional font (Consolas). Forty-eight participants viewed stimuli on a BenQ XL2410T LCD monitor while the remaining 72 viewed stimuli on a Cambridge Research Systems LCD++ monitor. Both monitors had a 1,920 × 1,080 resolution. To account for differences in pixel size between the two monitors, text was displayed in different font sizes: BenQ: 20 pt, Cambridge Research Systems: 16 pt. This ensured that at a viewing distance of 80 cm, 3.57 letters equated to 1° of visual angle. A forehead rest was used to minimise head movements and a VPixx five-button response box was used to record responses.

### Materials

**Figure 1.**
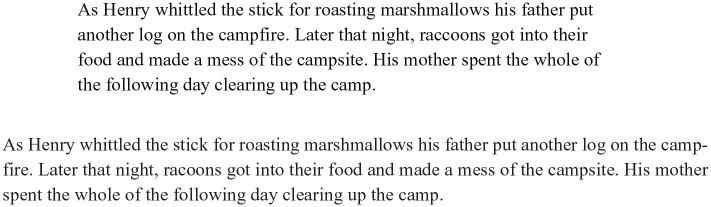
Example stimuli, where stimuli are formatted to one of two line lengths (75 or 115 characters).

Experimental stimuli consisted of 20 passages of text. Each passage contained three to six sentences displayed across three to four lines (see Figure 1), which were formatted to one of two line widths: 75 characters (21° of visual angle) or 115 characters (32° of visual angle). Words in the text varied in length from 1 to 12 letters (*M* = 4.35) and had an average Zipf’s frequency (Van Heuven et al., 2014) based on the SUBTLEX database (Brysbaert & New, 2009) of 5.80 (range: 1.30–7.67). Passages were counterbalanced so that each participant read an equal number in each condition and over all participants each passage was seen an equal number of times in each condition.

### Ability measures

#### Reading ability

To estimate effective reading rate, participants read three 120-word passages with an average word length of 5.07 characters, and a Flesch–Kincaid grade level of 9. After reading each passage, participants were required to answer 10 factually true-or-false questions about the passage. Participants’ effective reading rate was calculated as reading speed (words per minute) multiplied by their comprehension accuracy (Rayner et al., 2015; Slattery & Yates, 2018). While this effective reading rate measure has been used in previous studies (Slattery & Yates, 2018; Yates & Slattery, 2019), it differs from the way that reading ability has been operationalised by other labs. Typically, reading ability is assessed using the reading comprehension subtest of the Nelson–Denny (Brown et al., 1993). While Andrews et al. (2020) report that reading rate yields weaker correlations with spelling ability than comprehension performance on the Nelson–Denny, the correlation between effective reading rate (which combines comprehension and reading rate) and spelling ability in the current study is in line with other eye movement experiments (as discussed in the “Results” section).

#### Spelling ability

Two measures of spelling ability were administered (Andrews & Hersch, 2010; Andrews & Lo, 2012; Veldre & Andrews, 2014). The spelling dictation test required participants to spell 20 low-frequency words from a list compiled by Burt and Tate (2002). Words were read aloud, alone, and in a sentence, by the experimenter. A participant’s score on this test was the number of words correctly spelled. The spelling recognition task was comprised of 88 items, half of which were spelled incorrectly. Participants were required to indicate whether words were spelled correctly or incorrectly. A participant’s score on this second test was 88 minus the number of unidentified misspelled words or misidentified correctly spelled words. Thus, scores could range from 0 (none correct) to 88 (all correct).

### Procedure

Participants provided informed consent and were familiarised with the equipment. They then completed a 9-point calibration and validation procedure. Errors above 0.4° of visual angle were repeated. Prior to viewing stimuli, a black 2° × 2° square, which coincided with the left side of the first letter in the stimulus, appeared on the screen. Once a stable fixation was detected in this area, the stimulus was presented. Presentation order was randomised and participants were instructed to read silently for comprehension. Comprehension questions appeared after the third of items. These “yes/no” questions required participants to respond by pressing one of two buttons on the response box. The average accuracy for the comprehension questions was 86%.^2^ Following experimental trials, participants completed the individual differences tests in the following order: reading comprehension, spelling dictation, and misspelled word recognition. Upon completion, participants received payment at a rate of £10 per hour or course credit as compensation.

## Results

### Measures of reading and spelling ability

Descriptive statistics for individual difference measures are shown in Table 1. Scores on the spelling dictation and recognition tasks were highly correlated, *r* = .82, *p* < .001. Thus, following Andrews and Hersch (2010), the two spelling measures were standardised and then averaged together to create the zSpell variable. The effective reading rate was standardised to create the zRead variable. Consistent with prior research, these variables were positively correlated, *r* = .39, *p* < .001. Despite using different reading ability measures, the correlation between zRead and zSpell was similar to those reported by Andrews and colleagues (e.g., Andrews & Hersch, 2010; Drieghe et al., 2019; Veldre & Andrews, 2014; Veldre et al., 2017), which ranged from .34 to .48.

**Table 1.** Descriptive statistics for the individual difference variables.

	Effective reading rate (words per minute)	Spelling dictation (maximum = 20)	Spelling recognition (maximum = 88)
*M*	128.1	9.6	70.1
*SD*	42.30	4.23	8.29
Range	45–256	0–20	47–88

### Eye movement measures

Fixations shorter than 80 ms, which were within one character of a previous or subsequent fixation, were combined with that fixation while all other fixations less than 80 ms or greater than 800 ms were excluded, leading to the removal of 0.01% of fixations. Trials in which there were five or more blinks during passage reading were also removed (0.01% of trials).

Eye movement data were analysed using linear mixed-effects models (LMMs), constructed using the lme4 package (version 1.1-21; Bates et al., 2015) in R (R Core Team, 2019). Each model included a fixed-effects coding for line length condition. The *contr.sum()* function from the base stats package was used to implement summed-to-zero contrasts for our experimental line length manipulation, such that the intercept corresponded to the grand mean of line length conditions and the fixed effects corresponded to a main effect. To examine the independent contributions of reading and spelling ability on eye movement data, zSpell and zRead were entered as separate predictors and were allowed to interact with experimental effects in the models. Initially, all models adopted a full random structure, treating both participants and items as random factors, with random intercepts and slopes (Barr et al., 2013). To conserve power lost to unnecessary complexity, we used a “parsimonious” backwards selection approach to model the random-effects structure (Bates et al., 2015). All numerical variables were centred prior to analysis. For all models, we report regression coefficients (*b*), standard error (*SE*), *t*-values, and *p*-values (computed using the lmerTest package, version 3.1-0; Kuznetsova et al., 2019).

### Return-sweep and corrective saccade parameters

We examined three saccade parameters: return-sweep launch position (the number of characters from the end of the line at which the return-sweep is launched), return-sweep landing position (the number of characters from the left margin of the new line), and the frequency of corrective saccades. These metrics were examined for 4,354 return-sweeps in the short condition and 2,542 in the long condition. Return-sweep and corrective saccade parameters are shown in Table 2.

**Table 2.** Descriptive statistics for return-sweep and corrective saccade parameters as a function of line length.

Line Length	Return-sweep launch position	Return-sweep landing position	Frequency of undersweep fixations (%)
Short	5.7 (2.99)	5.4 (2.89)	63.3 (48.22)
Long	5.8 (3.00)	8.0 (3.46)	83.2 (37.4)

*Note.* Return-sweep launch sites are shown as the characters from the end of a line. Landing site is given in characters from the beginning of the line. Means are displayed with standard deviations in parenthesis.

**Figure 2.**
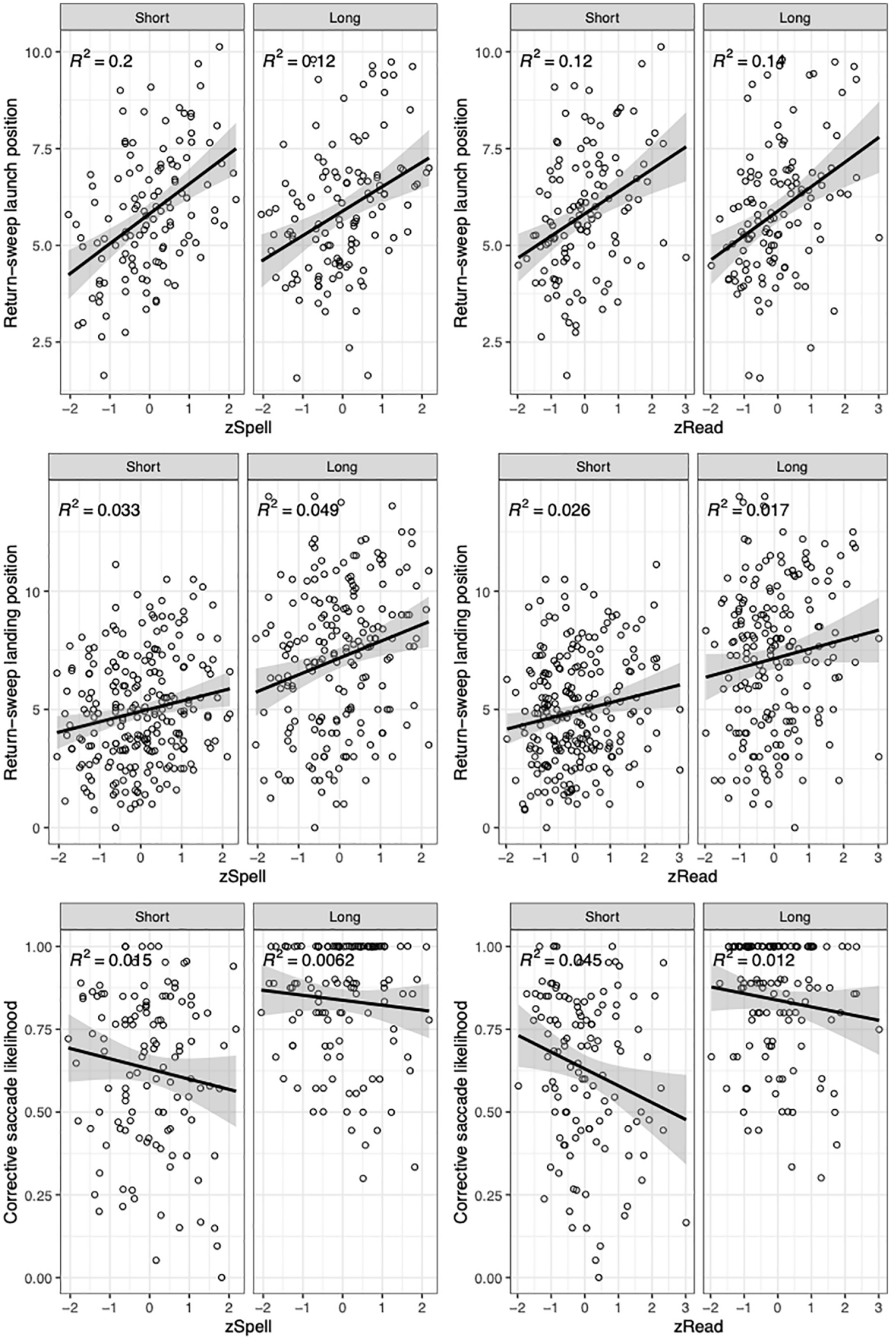
Scatterplots for dependent variables as a function of line length, zSpell, and zRead. Return-sweep launch position is measured in characters from the end of the line. Return-sweep landing position is measured in characters from the start of the line. Data points represent participants’ average score for each dependent variable correlated with their ability measures. The black line represents the regression line between the two variables. The grey band represents 95% confidence intervals.

First, we fitted an LMM to return-sweep launch position data: *lmer*(*dv~ condition* × *zSpell* + *condition* × *zRead* + (1| *participant*) + (1 + *condition| item*)). Prior to analysis, to exclude the extended right tail, we removed return-sweeps that were launched further than 20 characters from the end of the line (9.01% of return-sweeps). Regression coefficients shown in Table 3 indicate that return-sweep launch position did not differ between line length conditions (see Figure 2). However, return-sweep launch position increased with both increasing zSpell and zRead, indicating that highly skilled spellers and readers launched their return-sweeps further from the end of the line. The higher level interactions did not modulate return-sweep launch position.

**Table 3.** Results of the (generalised) linear mixed-effects models for return-sweep and corrective saccade parameters.

Measure	Fixed effects	*b*	*SE*	*t/z*	*p*
Return-sweep launch position	(Intercept)	**5.838**	**0.226**	**25.82**	**<.001**
	Condition	–0.017	0.104	–0.16	.870
	zSpell	**0.530**	**0.145**	**3.65**	**<.001**
	zRead	**0.435**	**0.139**	**3.13**	.**002**
	Condition × zSpell	0.073	0.042	1.74	.083
	Condition × zRead	–0.061	0.041	–1.49	.136
Return-sweep landing position	(Intercept)	**6.916**	**0.203**	**34.09**	**<.001**
	Condition	**–1.353**	**0.112**	**–12.03**	**<.001**
	zSpell	**0.414**	**0.191**	**2.17**	.**030**
	zRead	0.157	0.183	0.86	.390
	Launch position	**–0.066**	**0.014**	**–4.78**	**<.001**
	Condition × zSpell	–0.066	0.041	–1.59	.111
	Condition × zRead	–0.035	0.040	–0.87	.384
Corrective saccade likelihood	(Intercept)	**1.988**	**0.161**	**12.36**	**<.001**
	Condition	–0.024	0.047	–0.51	.613
	zSpell	**–0.385**	**0.165**	**–2.33**	.**020**
	zRead	**–0.425**	**0.158**	**–2.69**	.**007**
	Landing position	**0.779**	**0.026**	**29.72**	**<.001**
	Condition × zSpell	0.100	0.052	1.91	.056
	Condition × zRead	–0.026	0.052	–0.50	.616

*Note.* Significant model terms are presented in bold.

Next, we fitted a model to return-sweep landing position data: *lmer*(*dv~ condition* × *zSpell* + *condition* × *zRead* + *launch position* + (1| *participant*) + (1 *+* *condition| item*). In this model, return-sweep launch position was included as a centred numerical predictor to control for the position of the preceding line-final fixation. Prior to analysis, we excluded fixations landing more than 15 characters away from the start of the line (4.6% of cases). The relationship between fixed effects and return-sweep landing position is illustrated in Figure 2. Return-sweeps landed further from the left margin in the long line condition and when launch positions were close to the end of the prior line. There was also a main effect of zSpell where landing positions were further from the left margin for better spellers but not better readers. The remaining interactive model terms did not affect return-sweep landing position. This indicates that the effects of zSpell and zRead were consistent across the line length conditions.

Subsequently, we fitted a generalised LMM to examine the likelihood of initiating a corrective saccade following a return-sweep. In addition to fixed-effects coding for condition, zSpell, zRead, and their interactions, we included landing position as a control variable in the model. Return-sweep landing position has been shown to heavily influence the likelihood of initiating a corrective saccade following a return-sweep (Parker, Nikolova, et al., 2019; Parker, Slattery, & Kirkby, 2019). The model *glmer*(*dv~ landing position* + *condition* × *zSpell* + *condition* × *zRead* + (1| *participants*) *+* (1| *items*)) indicated that the likelihood of initiating a corrective saccade increased as a function of landing site. That is, return-sweeps landing further from the left margin were more likely followed by a corrective saccade. As shown in Figure 2, the likelihood of requiring a corrective saccade decreased with increasing zSpell and zRead. However, there was no statistically significant effect of line length condition or its interaction with zSpell or zRead on corrective saccade likelihood.^3^

### Return-sweeps and fixation duration

**Figure 3.**
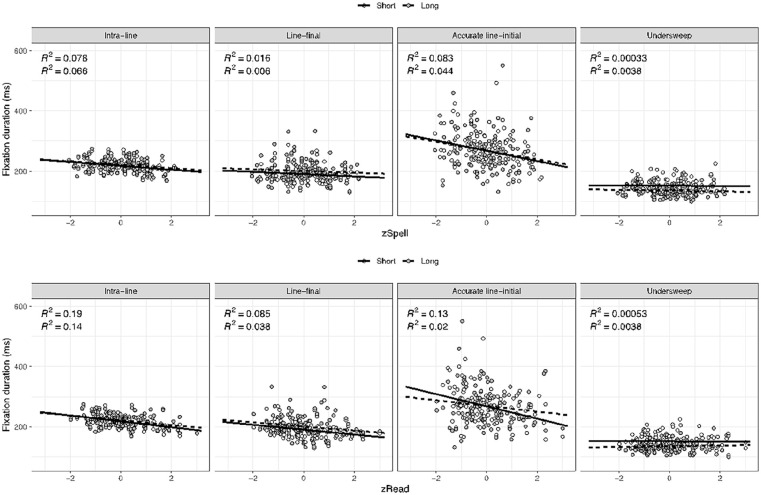
Scatterplots for fixation duration as a function of line length, zSpell, and zRead for each fixation population. Data points present participants’ average fixation duration correlated with their ability measures. The solid black line represents the regression line for the short line condition and the dashed line represents the regression line for the long condition. *R*^2^ values for the short line condition are shown above *R*^2^ values for the long line condition.

To assess how intra-line and return-sweep fixation durations differed with regard to spelling and reading ability, we fitted LMMs to log-transformed intra-line, line-final, accurate line-initial, and undersweep fixation durations: *lmer*(*dv~ condition* × *zSpell* + *condition* × *zRead* + (1 + *condition| participant)* + (1| *items*)). Mean durations for each fixation population are shown in Table 4. Regression coefficients in Table 5 indicated that, for intra-line fixations, durations were shorter in the short line condition. While intra-line fixation durations did not vary as a function of zSpell, they decreased with increasing zRead (see Figure 3). The significant interaction between condition and zRead suggested that the effect of zRead was stronger in the short line condition. Line-final fixations were shorter in the short line condition and decreased with increasing zRead, but not zSpell. While accurate line-initial fixation durations did not differ between line length conditions, they decreased with increasing zRead and zSpell. For accurate line-initial fixations, the effect of zRead on fixation duration is consistent with the large body of evidence indicating that better readers require less time for lexical access (Drieghe et al., 2019; Slattery & Yates, 2018; Veldre & Andrews, 2014, 2015; Veldre et al., 2017). However, the effect of zSpell on fixation duration for this specific fixation population is novel. Typically, spelling ability has been associated with longer forward saccades (parafoveal processing) rather than shorter fixation duration. So, this zSpell finding is new and consistent with spelling ability influencing early letter encoding processes rather than being specifically tied to parafoveal processing. Undersweep fixation durations were significantly shorter in the long line condition, yet did not vary as a function of zSpell or zRead, further supporting the notion that these fixations are not terminated based on lexical processing.

**Table 4.** Descriptive statistics for return-sweep fixation durations as a function of line length.

Line length	Intra-line	Line-final	Accurate line-initial	Undersweep
Short	200.6 (86.57)	191.4 (86.97)	257.9 (91.46)	147.9 (40.20)
Long	223.9 (86.81)	201.3 (94.81)	267. 3 (97.57)	134.3 (35.79)

*Note.* Means are displayed with standard deviations in parenthesis.

**Table 5.** Linear mixed-effects model results for fixation population duration (*log-ms*).

Fixation population	Fixed effect	*b*	*SE*	*t/z*	*p*
Intra-line	(Intercept)	**2.318**	**0.003**	**663.83**	**<.001**
	Condition	**–0.004**	**0.001**	**–6.09**	**<.001**
	zSpell	–0.006	0.004	–1.47	.141
	zRead	**–0.014**	**0.004**	**–3.99**	**<.001**
	Condition × zSpell	–0.000	0.001	–0.35	.730
	Condition × zRead	**–0.002**	**0.001**	**–2.36**	.**018**
Line-final	(Intercept)	**2.255**	**0.006**	**408.07**	**<.001**
	Condition	**–0.010**	**0.002**	**–4.62**	**<.001**
	zSpell	–0.002	0.006	–0.32	.747
	zRead	**–0.014**	**0.005**	**–2.68**	.**007**
	Condition × zSpell	–0.000	0.002	–0.17	.869
	Condition × zRead	–0.002	0.002	–0.67	.503
Accurate line-initial	(Intercept)	**2.400**	**0.007**	**324.43**	**<.001**
	Condition	–0.006	0.004	–1.66	.097
	zSpell	**–0.018**	**0.008**	**–2.30**	.**022**
	zRead	**–0.016**	**0.007**	**–2.17**	.**030**
	Condition × zSpell	0.003	0.004	0.64	.524
	Condition × zRead	–0.007	0.004	–1.65	.098
Undersweep	(Intercept)	**2.143**	**0.005**	**402.63**	**<.001**
	Condition	**0.023**	**0.001**	**15.85**	**<.001**
	zSpell	–0.005	0.006	–0.84	.399
	zRead	0.002	0.005	0.42	.675
	Condition × zSpell	0.001	0.002	0.84	.401
	Condition × zRead	–0.002	0.002	–1.54	.125

*Note.* Significant model terms are presented in bold.

## Discussion

The primary goal of this research was to characterise the effects of individual differences related to lexical quality on eye movement control during the reading of multiline texts, with particular emphasis on return-sweep saccades and their adjacent fixations. In addition to replicating several return-sweep findings, we report novel skill-related differences in relation to return-sweeps and corrective saccades. Less-skilled readers and spellers launched their return-sweeps closer to the end of the line, preferred a viewing location closer to the start, and made more return-sweep undershoot errors—as indexed by more undersweep fixations. We additionally report several skill-related differences in readers’ fixations across multiline texts. While reading skill influenced intra-line, line-final, and accurate line-initial fixations, undersweep fixations were not influenced by reading skill differences. In addition to being influenced by reading ability, accurate line-initial fixation durations were influenced by spelling ability. This is of particular interest as spelling ability is typically associated with measures of saccade targeting and oculomotor decisions as opposed to fixation durations. This suggests that spelling ability influences fixation durations when words are processed solely in foveal vision. Together, these results indicate that lexical quality not only shapes the rate at which readers access meaning from the text but influences the saccade targeting even in the absence of parafoveal information. We say more about these contributions with reference to our predictions below.

### Return-sweep and corrective saccade parameters

Consistent with our predictions, readers with higher quality lexical representations (as indexed by increased spelling and reading ability) launched their return-sweeps further from the end of the line. This indicates that those with more robust representations do not need to fixate the extremes of lines to encode the text there. This pattern of results parallels that of Parker, Slattery, and Kirkby (2019) whereby skilled, adult readers launched their return-sweeps further from the end of the line when compared with less-skilled, developing readers. Two potential reasons for these observations exist. The first is that more skilled readers are able to use parafoveal processing to encode the information presented in these extreme regions. This, of course, does not necessarily mean that better readers/spellers have larger perceptual spans. It may be that highly skilled readers engage in more efficient orthographic processing for information in near foveal areas that fall within the perceptual span (i.e., parafoveal regions of the word identification span—the area of the visual field in which words can be identified during a single fixation). The second is that skilled readers avoid fixating close to the end of a line to reduce oculomotor error associated with longer return-sweep saccades. This riskier saccade targeting may then result in readers guessing the identity of the line-final words to support ongoing lexical processing and comprehension. This strategy may be similar to the risky reading strategy seen in older adults where an increased reliance on top-down information from sentence context results in increased word skipping (Rayner et al., 2006). However, without empirical research on return-sweep execution in older adults, it remains difficult to draw these comparisons. In light of the current study, this second risky reading account seems unlikely. If skilled readers avoid fixating extreme positions, then we would predict an interaction between line length and ability whereby better readers and spellers launch their return-sweeps further from the end of the line when line length is long. This would prevent the execution of very long return-sweeps. Yet, the current data do not support this prediction. It instead appears that, regardless of line length, readers progress to a position from which they can adequately encode the line final letters.

Previous investigations have typically reported that return-sweep landing position is shifted to the right for longer lines (Heller, 1982; Hofmeister et al., 1999). In line with previous work and our predictions, such an effect was observed. In addition, skill-related differences were also observed for return-sweep landing positions. This is of particular interest as the target of the return-sweep lies far outside parafoveal vision. That is, even the most proficient readers and spellers would be unable to encode the letters at the start of a new line during the final fixation on the prior line.^4^ Instead, similar to the data reported by Parker, Slattery, and Kirkby (2019), highly skilled readers and spellers appear to have unconsciously learned to target further into lines. We believe that a shift in return-sweep targeting occurs over time as a reader develops more precise orthographic knowledge, and the ability to encode this orthographic information in the parafovea. Given that the shift in landing position with increasing spelling ability is small, it is likely that the development of precise orthographic knowledge promotes processing for near foveal information as opposed to information far in the parafovea/periphery. Nonetheless, these developments allow a reader to target further into a line safe in the knowledge that they will still be able to encode the letters at the start of the line. It is, however, important to reconcile our interpretation here with existing data. Veldre and Andrews (2014) reported that while spelling ability influenced the rightwards span, it did not influence the leftward span. So, at first glance, it may seem that our interpretation is inconsistent with the published literature. However, it is important to note that their study detailed single-sentence reading where attention is almost exclusively allocated from left to right. With return-sweeps, attention will be moving from right to left to execute the saccade as movement of attention precedes the saccade (Godijn & Pratt, 2002; Godijn & Theeuwes, 2003). Thus, the typical distribution of attention during reading cannot be assumed in this case. Indeed, when readers make an undersweep fixation, they appear to extract information from the line-initial word that facilities encoding of that word following a corrective saccade. Furthermore, there appears to be no such benefit for words to the right of an undersweep fixation, suggesting that the skew of attention is directed towards the start of the line following a return-sweep (c.f. Parker et al., 2020). One question that remains unanswered is the extent to which the distribution of attention changes during return-sweeps.

These observed skill-related effects in launch and landing positions highlight how ability differences acquired through learning can shape the reading strategy and can be interpreted within the context of Grainger and Ziegler’s (2011) dual-route approach to orthographic processing. Their approach hypothesises that two kinds of prelexical orthographic codes are used to identify words: a coarse-grained and a fine-grained code. Processing of coarse-grained information is rapid and promotes access to meaning. Within this route, a minimal subset of letters is used to identify a word. By contrast, the fine-grained route is dependent on precise letter order and word beginnings and endings. We propose that a skill-related shift in orthographic knowledge influences return-sweep targeting. It is possible that the increase in orthographic knowledge associated with high spelling and reading ability enables readers to rely less on fine-grained processing, and instead begin to use the most visible letters to “guess” the word’s identity. Thus, when engaged in this coarse route of processing, skilled readers may not have to fixate certain words at extreme positions on a line to process them. They can instead use the available information in parafoveal vision to do this.

What is perhaps most interesting in relation to skill-related differences is that poorer readers and spellers were more likely to require corrective saccades following a return-sweep despite their line-initial fixations generally landing closer to the left margin. Prior research has consistently shown that return-sweeps which land closer to the left margin are less likely to elicit a corrective saccade (Parker, Slattery, & Kirkby, 2019). Therefore, the closer landing sites combined with a greater likelihood of initiating corrective saccades for lower ability readers indicates the difficulty they are having processing extrafoveal information during a line-initial fixation. Furthermore, while a comparison of means indicated a higher frequency of corrective saccades in the long line condition, analysis indicated that line length itself did not predict the likelihood of initiating a corrective saccade. Instead, this was predicted only by the distance at which the reader landed relative to the start of the line (which was further away from the margin with long lines). This provides evidence to suggest that corrective saccades are executed based on information available following a return-sweep rather than being pre-planned with the return-sweep. Thus, it appears that the landing position of a return-sweep would influence the relationship between line length and corrective saccade likelihood that has been frequently reported (Beymer, Russel, & Orton, 2005; Dyson & Kipping, 1998; Heller, 1982; Paterson & Tinker, 1940; Schneps et al., 2013; Tinker, 1963). This interpretation is consistent with Findlay and Walker’s (1999) model of saccade generation that assumes saccades are generated during the preceding fixation. However, it does not rule out the possibility that readers are more prepared to initiate a corrective saccade following a return-sweep.

### Ability and fixation durations

While spelling and reading ability jointly influenced both return-sweep launch positions and corrective saccade likelihood, the duration of readers’ fixations were, for the most part, modulated by reading but not spelling ability. Intra-line fixation durations were exclusively modulated by reading ability. This observation replicates several prior eye movement studies (Drieghe et al., 2019; Slattery & Yates, 2018; Veldre & Andrews, 2015, 2016; Veldre et al., 2017). Line-final fixation durations were also exclusively influenced by reading ability, whereas undersweep fixation durations were not significantly influenced by reading or spelling ability. However, accurate line-initial fixations were influenced by both reading ability and spelling ability. These findings indicate that when parafoveal processing is possible prior to direct fixation (as is the case for intra-line and line-final fixations), the precise lexical representations measured by high spelling ability enhance the parafoveal processing that contributes to oculomotor decisions. When parafoveal processing is not possible during the prior fixation (as is the case for accurate line-initial fixations), spelling ability modulates the duration of the fixation itself.

Assuming that line-final fixations were primarily involved in return-sweep planning, we predicted that lexical quality may not modulate the duration of these fixations to the same extent as it would for intra-line fixations. However, line-final fixations decreased with increasing reading ability with similar estimates for zRead in both models. We see two possible explanations for this. First, if line-final fixations are involved in foveal encoding then better readers may complete this encoding faster, allowing them to execute return-sweeps earlier than poor readers. Second, if line-final fixations are strictly involved in return-sweep planning, better readers may take less time to plan and execute a return-sweep. Of course, it is entirely plausible that the effect of reading ability on line-final fixation duration reflects a mixture of the two explanations.

As we predicted, accurate line-initial fixations significantly decreased with increasing reading ability consistent with the importance of foveal word processing during these fixations (c.f. Parker & Slattery, 2019). We also observed an effect of spelling ability on accurate line-initial fixations, whereby durations significantly decreased with increasing spelling ability. This may seem puzzling given prior reports that the influence of spelling ability on eye movements during reading is exclusively restricted to word skipping and components associated with oculomotor targeting (Drieghe et al., 2019). Yet, it is important to note that these studies have involved intra-line reading where readers routinely have access to parafoveal information about a word prior to direct fixation. In the case of line-initial fixations, readers do not have access to this information prior to direct fixation. To compensate for this lack of information, readers may employ the precise lexical representations indexed by spelling ability. We see two possible ways in which this may occur. First, assuming that the duration of readers’ line-initial fixations is related to programming saccades across the line (i.e., start-up effects; Kuperman et al., 2010), then the precise lexical representations measured by spelling ability may influence the planning of these saccades with highly skilled spellers completing these plans more rapidly. Alternatively, the influence of spelling ability on reading processes may be to speed initial orthographic encoding. Since this encoding usually occurs parafoveally for adult readers, spelling ability’s influence is normally on measures of saccade length and word skipping, as better spellers are able to encode a greater number of letters parafoveally. With accurate line-initial fixations, this initial orthographic encoding will not have happened prior to direct fixation and therefore spelling ability will influence the duration of these fixations. Further work is required to confirm why spelling ability exerts an influence on this specific population of fixations.

In contrast to influences of zRead and zSpell on accurate line-initial fixations, undersweep fixation durations appear uninfluenced by reading or spelling ability. Undersweep fixation durations were approximately 142 ms, which is encompassed by the window (140–145 ms) at which lexical variables begin to influence fixation durations (Reingold & Sheridan, 2018). Despite this, neither reading nor spelling ability influenced the duration of undersweep fixations. That is not to say readers do not acquire information during these fixations. Slattery and Parker (2019), Parker and Slattery (2019), and Parker et al. (2020) reported that readers can extract information at the point of, and to the left of, undersweep fixations. However, these fixations appear to be terminated based on retinal feedback following the return-sweep which, if exceeding a certain threshold, triggers a corrective saccade. When this error is large, the corrective saccade will be initiated quicker than if the return-sweep landed closer to the start of the line (e.g., Becker, 1976). The current study provides evidence in support of this explanation as undersweep fixations landed further from the start of the line in the long line condition and were of a shorter duration relative to those in the short line condition. An absence of skill-related differences for undersweep fixation durations suggests that under certain circumstances, the oculomotor system drives fixation behaviour rather than lexical processing.

Before moving on to our concluding remarks, it is important to note a limitation of the current experiment. A potential confound here results from the line length manipulation. Given that the content of the texts were identical between conditions, the line length manipulation meant that words could occur in different spatial locations between conditions. Differences in lexical properties of the text in these locations could have systematically influenced return-sweep behaviour in these locations. As with prior work, this could have influenced saccade targeting between conditions. While this may have influenced return-sweep behaviour related to the line length manipulation, it is difficult to see how this may have affected the reading and spelling ability effects that were found and which did not interact with the line length manipulation. Still, future work should maintain consistency in the information presented to readers at the locations in which return-sweep launch sites and landing positions are most likely. For recent examples that have maintained consistency in the information presented to readers in these spatial locations while manipulating line length, see Parker, Nikolova, et al. (2019) and Vasilev, Adedeji et al. (2019).

In sum, the current work demonstrates that lexical quality, as indexed by reading and spelling ability, influences reading at line boundaries. Better readers require less time to encode information either side of the return-sweep, while better spellers use their precise lexical representations to more quickly encode information following a return-sweep. Better readers and spellers seem able to use parafoveal information to their advantage and do not need to target words positioned at extreme locations at the end of a line of text. What is perhaps more interesting is the observation that better spellers avoid fixating locations close to the start of the line despite being unable to process that information parafoveally on the prior fixation. This indicates that these better spellers target their return-sweeps based on the knowledge that they will be able to engage in parafoveal processing to the left of fixation following return-sweep execution. The observed skill-related differences in return-sweep behaviour are highly similar to the developmental differences between children and adults reported elsewhere (Parker et al., 2019). The distinctions between intra-line effects of reading and spelling ability are consistent with the growing body of research which suggests that reading ability primarily influences foveal processing while spelling ability influences parafoveal processing. However, this study is the first to establish that fixation durations are influenced by spelling ability when readers cannot engage in parafoveal processing prior to direct fixation on a word. This suggests that spelling skill may influence initial orthographic coding rather than parafoveal processing per se. These findings provide several benchmarks for computational models of eye movement control, such as E-Z Reader (Reichle & Sheridan, 2015) and SWIFT (Engbert et al., 2005). Currently, these models do not make predictions about return-sweep behaviour. To model the reading of larger passages of text, these models of eye movement control will have to consider fixation durations either side of a return-sweep and the factors contributing to return-sweep targeting and error.

## Notes

1. Measures of proficiency (i.e., zSpell, zRead) for participants whose left eye was tracked did not statistically differ from those whose right eye was tracked, nor did any return-sweep or corrective saccade parameter.

2. To examine how comprehension varied with spelling and reading ability, we fit a generalized linear mixed model (GLMM) to comprehension accuracy data: *glmer*(*dv~ zSpell* × *zRead* + (1| *participant*) + (1| *item*)). The model indicated that neither spelling ability, *b* = –0.135, *SE* = 0.172, *z* = –0.78, *p* = .433, nor reading ability, *b* = 0.067, *SE* = 0.156, *z* = 0.43, *p* = .665, influenced comprehension accuracy.

3. Without controlling for landing position, there was a significant effect of condition, *b* = –0.576, *SE* = 0.036, *t* = –16.01, *p* < .001, on corrective saccade likelihood, yet no effect of zSpell, *b* = –0.060, *SE* = 0.104, *t* = –0.57, *p* = .568, or zRead, *b* = –0.189, *SE* = 0.100, *t* = –1.90, *p* = .058. The interaction terms did not significantly affect corrective saccade likelihood (|*t|*s < –1.46). These results indicate that the effects of reading ability emerge only when we control for the position in which the return-sweep lands.

4. While this is true of most lines of text, we acknowledge that occasionally the line break between paragraphs will require a much shorter return-sweep to move gaze from the end of a partial line of text to the beginning of an indented line of text.

## References

[bibr1-1747021820949150] Andrews S. (2012). Individual differences in skilled word recognition and reading: The role of lexical quality. In Adelman J. (Ed.), Visual word recognition (Vol. 2, pp. 151–172). Psychology Press.

[bibr2-1747021820949150] Andrews S. Hersch J. (2010). Lexical precision in skilled readers: The role of lexical quality. Individual differences in masked neighbour priming. Journal of Experimental Psychology: General, 139, 299–318. 20438253 10.1037/a0018366

[bibr3-1747021820949150] Andrews S. Lo S. (2012). Not all skilled readers have cracked the code: Individual differences in masked form priming. Journal of Experimental Psychology: Learning, Memory, and Cognition, 38, 152–163. 10.1037/a0024953 21875252

[bibr4-1747021820949150] Andrews S. Veldre A. Clarke I. E. (2020). Measuring lexical quality: The role of spelling ability. Behavior Research Methods. Advance online publication. 10.3758/s13428-020-01387-3 32291733

[bibr5-1747021820949150] Ashby J. Rayner K. Clifton C. (2005). Eye movements of highly skilled and average readers: Differential effects of frequency and predictability. Quarterly Journal of Experimental Psychology Section A, 58, 1065–1086. 10.1080/02724980443000476 16194948

[bibr6-1747021820949150] Barr D. J. Levy R. Scheepers C. Tily H. J. (2013). Random effects structure for confirmatory hypothesis testing: Keep it maximal. Journal of Memory and Language, 68, 255–278. 10.1016/j.jml.2012.11.001 PMC3881361 24403724

[bibr7-1747021820949150] Bates D. Kliegl R. Vasishth S. Baayen H. (2015). Parsimonious mixed models. arXiv preprint arXiv: 1506 04967.

[bibr8-1747021820949150] Bates D. Maechler M. Bolker B. Walker S. (2015). Fitting linear mixed-effects models using lme4. Journal of Statistical Software, 67, 1–48.

[bibr9-1747021820949150] Becker W. (1976). Do correction saccades depend exclusively on retinal feedback? A note on the possible role of non-retinal feedback. Vision Research, 16, 425–427. 941420 10.1016/0042-6989(76)90209-1

[bibr10-1747021820949150] Bélanger N. N. Slattery T. J. Mayberry R. I. Rayner K. (2012). Skilled deaf readers have an enhanced perceptual span in reading. Psychological Science, 23, 816–823. 22683830 10.1177/0956797611435130 PMC3723350

[bibr11-1747021820949150] Beymer D. Russell D. M. Orton P. Z. (2005). Wide vs. narrow paragraphs: An eye tracking analysis. In Costabile M. F. Paternò F. (Eds.) IFIP Conference on Human-Computer Interaction (pp. 741–752). Springer.

[bibr12-1747021820949150] Brown J. I. Fishco V. V. Hanna G. (1993). Nelson-Denny Reading Test. Riverside Publishing Company.

[bibr13-1747021820949150] Brysbaert M. New B. (2009). Moving beyond Kučera and Francis: A critical evaluation of current word frequency norms and the introduction of a new and improved word frequency measure for American English. Behavior Research Methods, 41, 977–990. 19897807 10.3758/BRM.41.4.977

[bibr14-1747021820949150] Burt J. S. Tate H. (2002). Does a reading lexicon provide orthographic representations for spelling? Journal of Memory and Language, 46, 518–543.

[bibr15-1747021820949150] Chace K. H. Rayner K. Well A. D. (2005). Eye movements and phonological parafoveal preview: Effects of reading skill. Canadian Journal of Experimental Psychology/Revue canadienne de psychologie expérimentale, 59, 209. 16248500 10.1037/h0087476

[bibr16-1747021820949150] Drieghe D. Veldre A. Fitzsimmons G. Ashby J. Andrews S. (2019). The influence of number of syllables on word skipping during reading revisited. Psychonomic Bulletin & Review, 26, 616–621. 30877634 10.3758/s13423-019-01590-0

[bibr17-1747021820949150] Dyson M. C. Kipping G. J. (1998). The effects of line length and method of movement on patterns of reading from screen. Visible Language, 32, 150–181.

[bibr18-1747021820949150] Engbert R. Nuthmann A. Richter E. M. Kliegl R. (2005). SWIFT: A dynamical model of saccade generation during reading. Psychological Review, 112(4), 777. 16262468 10.1037/0033-295X.112.4.777

[bibr19-1747021820949150] Findlay J. M. Walker R. (1999). A model of saccade generation based on parallel processing and competitive inhibition. Behavioral and Brain Sciences, 22, 661–674. 10.1017/s0140525x99002150 11301526

[bibr20-1747021820949150] Godijn R. Pratt J. (2002). Endogenous saccades are preceded by shifts of visual attention: Evidence from cross-saccadic priming effects. Acta Psychologica, 110(1), 83–102. 12005230 10.1016/s0001-6918(01)00071-3

[bibr21-1747021820949150] Godijn R. Theeuwes J. (2003). The relationship between exogenous and endogenous saccades and attention. In Hyönä J. Radach R. Deubel H. (Eds.), The mind’s eye (pp. 3–26). North-Holland.

[bibr22-1747021820949150] Grainger J. Ziegler J. (2011). A dual-route approach to orthographic processing. Frontiers in Psychology, 2, 54. 21716577 10.3389/fpsyg.2011.00054 PMC3110785

[bibr23-1747021820949150] Heller D. (1982). Eye movements in reading. In Groner R. Fraisse P. (Eds.), Cognition and eye movements (pp. 487–498). Deutscher Verlag der Wissenschaften.

[bibr24-1747021820949150] Hofmeister J. (1997). On corrective saccades in reading and reading-like tasks [Unpublished doctoral dissertation, Rheinisch-Westfälische Technische Hochschule].

[bibr25-1747021820949150] Hofmeister J. Heller D. Radach R. (1999). The return sweep in reading. In Becker W. Deubel H. Mergner T. (Eds.), Current oculomotor research: Physiological and psychological aspects (pp. 349–357). Plenum Press.

[bibr26-1747021820949150] Jared D. Levy B. A. Rayner K. (1999). The role of phonology in the activation of word meanings during reading: Evidence from proofreading and eye movements. Journal of Experimental Psychology: General, 128, 219–264. 10513396 10.1037//0096-3445.128.3.219

[bibr27-1747021820949150] Kuperman V. Dambacher M. Nuthmann A. Kliegl R. (2010). The effect of word position on eye-movements in sentence and paragraph reading. Quarterly Journal of Experimental Psychology, 63, 1838–1857. 10.1080/17470211003602412 20373225

[bibr28-1747021820949150] Kuperman V. Van Dyke J. A. (2011). Effects of individual differences in verbal skills on eye-movement patterns during sentence reading. Journal of Memory and Language, 65, 42–73. 21709808 10.1016/j.jml.2011.03.002 PMC3119501

[bibr29-1747021820949150] Kuznetsova A. Brockhoff P. B. Christensen R. H. B. (2017). lmerTest package: Tests in linear mixed effects models. Journal of Statistical Software, 82, 1–26.

[bibr30-1747021820949150] McGowan V. A. Reichle E. D. (2018). The “risky” reading strategy revisited: New simulations using EZ Reader. Quarterly Journal of Experimental Psychology, 71(1), 179–189. 10.1080/17470218.2017.1307424 PMC6143430 28426352

[bibr31-1747021820949150] Netchine S. Guihou M. C. Greenbaum C. Englander G. (1983). Retour à la ligne, âge des lecteurs et accessibilité au texte. Le Travail Humain [Back to the line, age of readers and accessibility to text], 46, 139–153.

[bibr32-1747021820949150] Parker A. J. Kirkby J. A. Slattery T. J. (2017). Predictability effects during reading in the absence of parafoveal preview. Journal of Cognitive Psychology, 29, 902–911.

[bibr33-1747021820949150] Parker A. J. Kirkby J. A. Slattery T. J. (2020). Undersweep fixations during reading in adults and children. Journal of Experimental Child Psychology, 192, e104788. 31981751 10.1016/j.jecp.2019.104788

[bibr34-1747021820949150] Parker A. J. Nikolova M. Slattery T. J. Liversedge S. P. Kirkby J. A. (2019). Binocular coordination and return-sweep saccades among skilled adult readers. Journal of Vision, 19, 1–10. 10.1167/19.6.10 31185092

[bibr35-1747021820949150] Parker A. J. Slattery T. J. (2019). Word frequency, predictability, and return-sweep saccades: Towards the modeling of eye movements during paragraph reading. Journal of Experimental Psychology: Human Perception and Performance, 45, 1614–1633. 31524433 10.1037/xhp0000694

[bibr36-1747021820949150] Parker A. J. Slattery T. J. Kirkby J. A. (2019). Return-sweep saccades during reading in adults and children. Vision Research, 155, 35–43. 30625336 10.1016/j.visres.2018.12.007

[bibr37-1747021820949150] Paterson D. G. Tinker M. A. (1940). How to make type readable. Harper.

[bibr38-1747021820949150] Perfetti C. A. (1992). The representation problem in reading acquisition. In Gough P. B. Ehri L. C. Treiman R. (Eds.), Reading acquisition (pp. 145–174). Lawrence Erlbaum.

[bibr39-1747021820949150] Perfetti C. A. (2007). Reading ability: Lexical quality to comprehension. Scientific Studies of Reading, 11, 357–383.

[bibr40-1747021820949150] Rayner K. Abbott M. J. Plummer P. (2015). Individual differences in perceptual processing and eye movements in reading. In Rayner K. Abbott M. J. Plummer P. (Eds.), Handbook of individual differences in reading: Reader, text, and context (pp. 348–363). Routledge.

[bibr41-1747021820949150] Rayner K. Reichle E. D. Stroud M. J. Williams C. C. Pollatsek A. (2006). The effect of word frequency, word predictability, and font difficulty on the eye movements of young and older readers. Psychology and Aging, 21, 448–465. 16953709 10.1037/0882-7974.21.3.448

[bibr42-1747021820949150] Rayner K. Slattery T. J. Bélanger N. N. (2010). Eye movements, the perceptual span, and reading speed. Psychonomic Bulletin & Review, 17, 834–839. 21169577 10.3758/PBR.17.6.834 PMC3075059

[bibr43-1747021820949150] R Core Team. (2019). R: A language and environment for statistical computing. R Foundation for Statistical Computing. https://www.R-project.org/

[bibr44-1747021820949150] Reichle E. D. Sheridan H. (2015). E-Z Reader: An overview of the model and two recent applications. In Pollatsek A. Treiman R. (Eds.), The Oxford handbook of reading (pp. 277–290). Oxford University Press.

[bibr45-1747021820949150] Reingold E. M. Sheridan H. (2018). On using distributional analysis techniques for determining the onset of the influence of experimental variables. Quarterly Journal of Experimental Psychology, 71, 260–271. 10.1080/17470218.2017.1310262 28430076

[bibr46-1747021820949150] Schneps M. H. Thomson J. M. Sonnert G. Pomplun M. Chen C. HeffnerWong A. (2013). Shorter lines facilitate reading in those who struggle. PLOS ONE, 8, e71161. 23940709 10.1371/journal.pone.0071161 PMC3734020

[bibr47-1747021820949150] Slattery T. J. Parker A. J. (2019). Return sweeps in reading: Processing implications of undersweep-fixations. Psychonomic Bulletin & Review, 26, 1948–1957. 31325039 10.3758/s13423-019-01636-3 PMC6863793

[bibr48-1747021820949150] Slattery T. J. Vasilev M. R. (2019). An eye-movement exploration into return-sweep targeting during reading. Attention, Perception, & Psychophysics, 81, 1197–1203. 10.3758/s13414-019-01742-3 PMC6647890 31165454

[bibr49-1747021820949150] Slattery T. J. Yates M. (2018). Word skipping: Effects of word length, predictability, spelling and reading skill. Quarterly Journal of Experimental Psychology, 71, 250–259. 10.1080/17470218.2017.1310264 PMC6159777 28856970

[bibr50-1747021820949150] Stanovich K. E. (2000). Progress in understanding reading: Scientific foundations and new frontiers. Guilford Press.

[bibr51-1747021820949150] Tinker M. A. (1963). Legibility of print. Iowa State University Press.

[bibr52-1747021820949150] Trauzettel-Klosinski S. Koitzsch A. M. Dürrwächter U. Sokolov A. N. Reinhard J. Klosinski G. (2010). Eye movements in German-speaking children with and without dyslexia when reading aloud. Acta Ophthalmologica, 88, 681–691. 19508458 10.1111/j.1755-3768.2009.01523.x

[bibr53-1747021820949150] Van Heuven W. J. Mandera P. Keuleers E. Brysbaert M. (2014). SUBTLEX-UK: A new and improved word frequency database for British English. Quarterly Journal of Experimental Psychology, 67, 1176–1190. 10.1080/17470218.2013.850521 24417251

[bibr54-1747021820949150] Vasilev M. R. Adedeji V. I. Laursen C. Budka M. Slattery T. J. (2019). Do readers use character information when programming return-sweep saccades? arXiv preprint arXiv: 1911 00716. 10.1016/j.visres.2021.01.003 33652273

[bibr55-1747021820949150] Vasilev M. R. Yates M. Slattery T. J. (2019). Do readers integrate phonological codes across saccades? A Bayesian meta-analysis and a survey of the unpublished literature. Journal of Cognition, 2, e43. 10.5334/joc.87 PMC6838770 31750415

[bibr56-1747021820949150] Veldre A. Andrews S. (2014). Lexical quality and eye movements: Individual differences in the perceptual span of skilled adult readers. Quarterly Journal of Experimental Psychology, 67, 703–727. 10.1080/17470218.2013.826258 23972214

[bibr57-1747021820949150] Veldre A. Andrews S. (2015). Parafoveal preview benefit is modulated by the precision of skilled readers’ lexical representations. Journal of Experimental Psychology: Human Perception and Performance, 41, 219–232. 25384238 10.1037/xhp0000017

[bibr58-1747021820949150] Veldre A. Drieghe D. Andrews S. (2017). Spelling ability selectively predicts the magnitude of disruption in unspaced text reading. Journal of Experimental Psychology: Human Perception and Performance, 43, 1612–1628. 28414501 10.1037/xhp0000425

[bibr59-1747021820949150] Yates M. Slattery T. J. (2019). Individual differences in spelling ability influence phonological processing during visual word recognition. Cognition, 187, 139–149. 30875660 10.1016/j.cognition.2019.02.015

